# Accelerating eye movement research via accurate and affordable smartphone eye tracking

**DOI:** 10.1038/s41467-020-18360-5

**Published:** 2020-09-11

**Authors:** Nachiappan Valliappan, Na Dai, Ethan Steinberg, Junfeng He, Kantwon Rogers, Venky Ramachandran, Pingmei Xu, Mina Shojaeizadeh, Li Guo, Kai Kohlhoff, Vidhya Navalpakkam

**Affiliations:** 1 Google Research, Mountain View, CA USA; 2 Present Address: Stanford University, Stanford, CA USA; 3 Present Address: Georgia Institute of Technology, Atlanta, GA USA; 4 Present Address: Johns Hopkins University, Baltimore, MD USA

**Keywords:** Attention, Visual system, Human behaviour

## Abstract

Eye tracking has been widely used for decades in vision research, language and usability. However, most prior research has focused on large desktop displays using specialized eye trackers that are expensive and cannot scale. Little is known about eye movement behavior on phones, despite their pervasiveness and large amount of time spent. We leverage machine learning to demonstrate accurate smartphone-based eye tracking without any additional hardware. We show that the accuracy of our method is comparable to state-of-the-art mobile eye trackers that are 100x more expensive. Using data from over 100 opted-in users, we replicate key findings from previous eye movement research on oculomotor tasks and saliency analyses during natural image viewing. In addition, we demonstrate the utility of smartphone-based gaze for detecting reading comprehension difficulty. Our results show the potential for scaling eye movement research by orders-of-magnitude to thousands of participants (with explicit consent), enabling advances in vision research, accessibility and healthcare.

## Abstract (web summary)

Progress in eye movement research has been limited since existing eye trackers are expensive and do not scale. Here, the authors show that smartphone-based eye tracking achieves high accuracy comparable to state-of-the-art mobile eye trackers, replicating key findings from prior eye movement research.

## Introduction

As we move through rich and complex environments in our everyday life, the retina is bombarded with vast amounts of visual information of ~10^10^ bits/s^1,2^. Selective attention is the mechanism by which our brain selects and focuses on a few important scene regions for cognitive and visual processing (see refs. ^3–5^). The human eye moves 3–4 times per second on average, pausing to sample information from those important scene regions^6–8^. Thus, eye movements offer a direct way to measure overt spatial attention, and have been considered by some to provide a window into the brain and mind^9,10^. Understanding eye movements has been central to research in attention and visual processing in the brain, including focus areas such as visual search^11–13^, scene perception^14–16^, and reading^17,18^, to name a few.

Beyond basic vision research, eye movements have also been of interest to the broader research community with applications ranging from saliency models for visual content analysis^19^, design evaluation^20^, usability and consumer behavior research^21–23^, driving^24^, gaming^25,26^, gaze-based interaction for accessibility^27^ to medical research^28,29^. The underlying methodology, known as eye tracking, has been used for decades as a reliable way to measure eye movements^30–32^.

Despite the numerous benefits of eye tracking, research and applications have been limited by the high cost of eye trackers and their inability to scale due to the use of specialized hardware (e.g., infrared light source, multiple high spatio-temporal resolution infrared cameras). There are some cheaper eye tracking solutions available for the desktop^33,34^, though not for mobile screens (state-of-the-art mobile eye trackers cost on the order of ten thousand USD). Further, little is known about eye movement behavior on small smartphone displays as most prior research focused on large desktop displays. Recent estimates show over 2.8 billion smartphone users worldwide^35^, with nearly twice as much time spent consuming content on mobile devices as desktop/laptop in the US (increases to 3× in India, 6× in China), and exceeding time spent watching TV^36^. Given their pervasiveness, accurate and affordable eye tracking on smartphones could enable significant advances in eye movement research by providing orders-of-magnitude scaling and generating insights across diverse populations, as well as unlocking applications across vision research, accessibility and healthcare.

Recent approaches in machine learning (ML) have shown promise for eye tracking using the existing front-facing cameras (selfie cameras) on smartphones^37,38^ and laptops^39,40^. However, their accuracy has been too low for rigorous eye movement research (2.56–3^∘^ for laptops^39,40^ and 2.44–3^∘^ viewing angle for smartphones^37,38^ compared to 0.5–1^∘^ for specialized eye trackers).

## Results

### Model accuracy

We trained a multi-layer feed-forward convolutional neural network (ConvNet). The model takes as input an RGB image from a smartphone’s front-facing camera cropped to the eye regions, and applies three layers of convolution to extract gaze features. The features are combined in additional layers with automatically-extracted eye corner landmarks indicating the eye position within the image for a final on-screen gaze estimate. This base model was first trained using the publicly available GazeCapture dataset^37^, then fine-tuned using calibration data and personalized by fitting an additional regression model (details in the “Methods” section) to the gaze feature output from the ConvNet, described below.

During calibration, participants were asked to fixate on a green circular stimulus that appeared on a black screen. The stimulus appeared at random locations on the screen. Images from the front-facing camera were recorded at 30 Hz and timestamps synchronized with the marker location. In ML terminology, images and marker locations served as inputs and targets, respectively. During inference, the camera images were fed in sequence to the fine-tuned base model whose penultimate layer served as input to the regression model to get the final, personalized gaze estimate. Model accuracy was evaluated across all participants by computing the error in cm between stimulus locations from the calibration tasks (ground truth) and the estimated gaze locations.

**Fig. 1:**
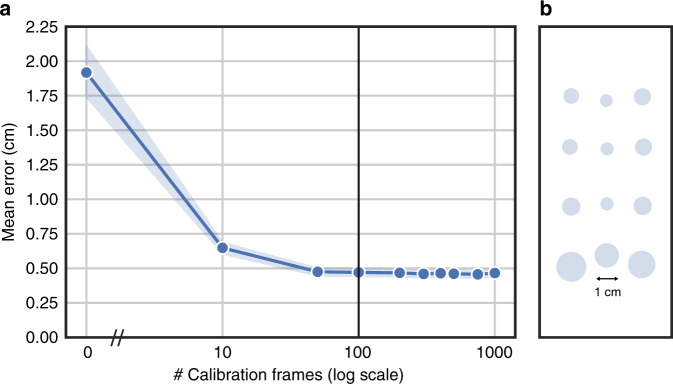
Accuracy of our smartphone eye tracker. **a** Gaze estimation accuracy (mean ± s.e.m., *n* = 26 participants) improves with # calibration frames for personalization. **b** Error across different screen locations. The radius of the circle indicates average model error at that screen location.

To test the effect of personalization on model accuracy, we collected data from 26 participants as they viewed stimuli on the phone, mounted on a device stand. Similar to typical eye tracking studies on the desktop, we focused on a near frontal headpose (no tilt/pan/roll; see “Methods”, study 1). Figure 1 shows how accuracy varies with the number of calibration frames. While the base model has a high error of 1.92 ± 0.20 cm, personalization with  ~100 calibration frames led to a nearly fourfold reduction in error resulting in 0.46 ± 0.03 cm (t(25) = 7.32, *p* = 1.13 × 10^−7^). Note that 100 calibration frames across different screen locations corresponds to  <30 s of data, which is quite reasonable for eye tracking studies where calibration is typically performed at the beginning of each study (or during the study to account for breaks or large changes in pose). The best participant had 0.23 cm error, while the worst participant had 0.75 cm error ([5,95]th percentiles were [0.31,0.72] cm). At a viewing distance of 25–40 cm, this corresponds to 0.6–1^∘^ accuracy, which is better than 2.44–3^∘^ for previous work^37,38^.

The improvements over previous work are due to a combination of better model architecture, calibration/personalization, and optimal UX settings. In particular, fine-tuning and personalizing the model using  ~30 s of calibration data under optimal UX settings (near frontal headpose, short viewing distance of 25–40 cm) led to big accuracy improvements (1.92–0.46 cm). While changes in model architecture led to modest improvements in accuracy (0.73 cm^37^ to 0.46 cm for ours, with fine-tuning and personalization applied to both models), they significantly reduced model complexity by 50× (8 M vs. 170 K model parameters), making it suitable for on-device implementation. Thus, our model is both lightweight and accurate.

As shown in Fig. 1b, the errors were comparable across different locations on the phone screen, with slightly larger error toward the bottom screen locations since the eyes tend to appear partially closed when participants look down (see Supplementary Fig. 1). While these numbers are reported for Pixel 2 XL phones, personalization was found to help across other devices as well (see Supplementary Fig. 3a). Figures 1a, b focused on the frontal headpose such that the face covered about one-third of the camera frame. To test the effect of headpose and distance on accuracy, we analyzed the GazeCapture^37^ dataset on iPhones, which offered more diversity in headpose/distance. As seen in Supplementary Figs. 3b–e, the best performance was achieved for near frontal headpose and shorter distance to the phone (where the eye region appeared bigger), and accuracy decayed with increasing pan/tilt/roll, or as participants moved further away from the phone. Thus, all studies in this paper focused on the optimal UX settings, namely near frontal headpose with short viewing distances of 25–40 cm to the phone. While this may seem restrictive, it is worth noting that the most common eye tracking setup for prior eye movement research^8,12,14,16,18,29^ often requires expensive hardware and more controlled settings such as chin rest with dim indoor lighting and fixed viewing distance.

### Comparison with specialized mobile eye trackers

**Fig. 2:**
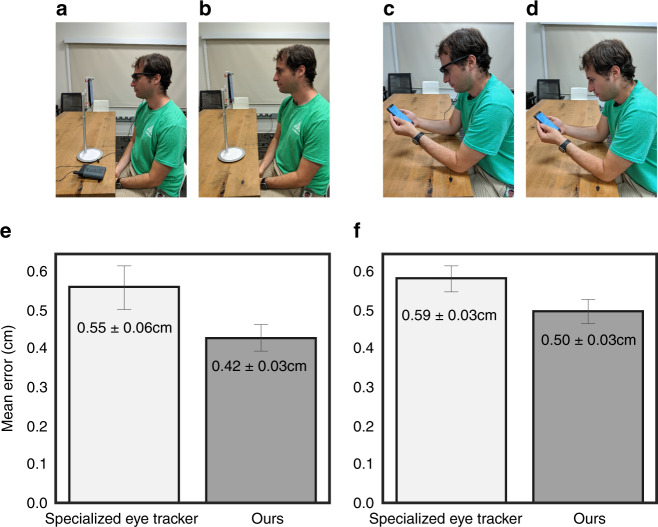
Comparison between accuracy of Tobii glasses vs. our model. Study setup shows the four experimental conditions: Participant (an author for visualization purposes) views stimuli on the phone (mounted on a device stand) while wearing Tobii glasses (**a**) and without (**b**). **c**, **d** Similar to the above, but participant holds the phone in the hand. **e**, **f** Accuracy of specialized eye tracker (Tobii glasses) vs. our smartphone eye tracker (mean ± s.e.m., *n* = 13 participants) for the device stand and hand-held settings. Statistical comparison shows no significant difference in accuracy across both settings (device stand: t(12) = −2.12, *p* = 0.06; hand-held: t(12) = −1.53, *p* = 0.15; two-tailed paired *t*-test).

To understand the gap in performance between our smartphone eye tracker and state-of-the-art, expensive mobile eye trackers, we compared our method against Tobii Pro 2 glasses which is a head mounted eye tracker with four infrared cameras near the eye. We selected the frontal headpose since Tobii glasses work best in this setting. Thirteen users performed a calibration task under four conditions—with and without Tobii glasses, with a fixed device stand and freely holding the phone in the hand (see Fig. 2). With the fixed device stand, we found that the smartphone eye tracker’s accuracy (0.42 ± 0.03 cm) was comparable to Tobii glasses (0.55 ± 0.06 cm, two-tailed paired *t*-test, t(12) = −2.12, *p* = 0.06). Similar results were obtained in the hand-held setting (0.59 ± 0.03 cm on Tobii vs. 0.50 ± 0.03 cm on ours; t(12) = −1.53, *p* = 0.15). The error distribution per user for both the device stand and hand-held settings can be found in Supplementary Fig. 4.

It is worth noting that specialized eye trackers like Tobii Pro glasses represent a high bar. These are head mounted glasses with four infrared cameras (two near each eye) and one world centered camera. Thus the input is high-resolution infrared images of close-up of the eyes (within 5–10 cm distance from the eye). In contrast, our method uses the smartphone’s single front-facing RGB camera, at larger viewing distance (25–40 cm from the eye), hence the eye region appears small. Despite these challenges, it is promising that our smartphone eye tracker achieves comparable accuracy as state-of-the-art mobile eye trackers.

### Validation on standard oculomotor tasks

**Fig. 3:**
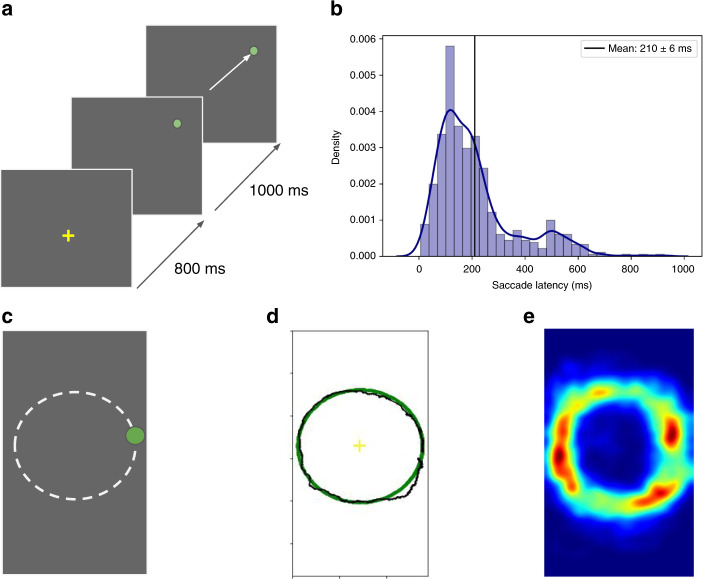
Smartphone gaze for standard oculomotor tasks. **a** Prosaccade task. Each trial began with a central fixation for 800 ms, after which the target appeared at a random location and remained for 1000 ms. Participants were asked to saccade to the target as soon as it appeared. **b** Saccade latency distribution for the prosaccade task. **c** Smooth pursuit task. Participants were asked to look at the green dot as it moved along a circle. **d** Sample scanpath from a single user shown in black (ground truth in green). **e** Population-level heatmap from all users and trials.

As a research validation, we tested whether the key findings from previous eye movement research on oculomotor tasks using large displays and expensive desktop eye trackers, could be replicated on small smartphone displays using our method. Twenty-two participants performed prosaccade, smooth pursuit and visual search tasks as described below (details in “Methods”, study 2). Figure 3a shows the setup for the prosaccade task. We computed saccade latency, a commonly studied measure, as the time from when the stimulus appeared to when the participant moved their eyes. As seen in Fig. 3b, mean saccade latency was 210 ms (median 167 ms), consistent with 200–250 ms observed in previous studies^41^.

To investigate smooth pursuit eye movements, participants were asked to perform two types of tasks—one where the object moved smoothly along a circle, and another along a box. Similar tasks have been recently demonstrated to be useful for detecting concussion^42,43^. Figures 3c–e show sample gaze scanpath from a randomly selected participant, and the population-level heatmap from all users and trials for the smooth pursuit circle task. Consistent with previous literature on desktops, participants performed well in this task, with a low tracking error of 0.39 ± 0.02 cm. Similar results were obtained for the smooth pursuit box task (see Supplementary Fig. 5).

Beyond simple oculomotor tasks, we investigated visual search which has been a key focus area of attention research since 1980s^12,44,45^. Two well-known phenomena here are: (1) the effect of target saliency (dissimilarity or contrast between the target and surrounding distracting items in the display, known as distractors)^46,47^; (2) and the effect of set size (number of items in the display)^44,45^ on visual search behavior.

**Fig. 4:**
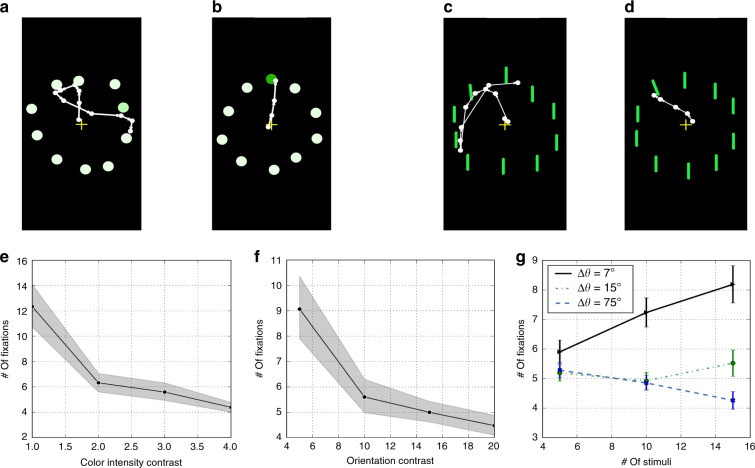
Smartphone gaze during visual search. **a**, **b**, **e** Effect of target’s color contrast on visual search performance. **a** Gaze scanpath when the target has low contrast (i.e., similar to the distractors). **b** Scanpath when the target has high contrast (different from the distractors). **e** Number of fixations to find the target as a function of target’s color contrast (plot shows mean ± s.e.m., *n* =  44–65 trials/contrast-level). **c**, **d**, **f** Similar plots for orientation contrast (difference in orientation between target and distractors in degrees, Δ*θ*; *n* = 42–63 trials/contrast-level). **g** Effect of set size. Number of fixations to find the target as the number of items in the display varied between 5, 10, and 15; and the target’s orientation contrast varied from low (Δ*θ* = 7^∘^) to medium-high (Δ*θ* = 15^∘^) to very high (Δ*θ* = 75^∘^). Plot shows mean ± s.e.m. in number of fixations (*n* = 42–63 trials for each combination of set size and Δ*θ*).

To test the presence of these effects on phones, we measured gaze patterns as 22 participants performed a series of visual search tasks. We systematically varied the target’s color intensity or orientation relative to the distractors. When the target’s color (or orientation) appeared similar to the distractors (low target saliency), more fixations were required to find the target (see Fig. 4a, c). In contrast, when the target’s color (or orientation) appeared different from the distractors (high target saliency), fewer fixations were required (Fig. 4b, d). We found that across all users and trials, the number of fixations to find the target decreased significantly as target saliency increased (see Fig. 4e, f for color intensity contrast: F(3, 63) = 37.36, *p* < 10^−5^; for orientation contrast: F(3, 60) = 22.60, *p* < 10^−5^). These results confirm the effect of target saliency on visual search, previously seen in desktop studies^12,44,46,47^.

To test the effect of set size on visual search, we varied the number of items in the display from 5, 10 to 15. Figure 4g shows that the effect of set size depends on target saliency. When the target saliency is low (difference in orientation between target and distractors, Δ*θ* = 7^∘^), the number of fixations to find the target increased linearly with set size (slope = 0.17; one-way repeated measures ANOVA F(2, 40) = 3.52, *p* = 0.04). In contrast, when the target saliency is medium-high (Δ*θ* = 15^∘^), the number of fixations to find the target did not vary significantly with set size (F(2, 40) = 0.85, *p* = 0.44). For very highly salient targets (Δ*θ* = 75^∘^), we found a negative effect of set size on the number of fixations (slope = −0.06; F(2, 40) = 4.39, *p* = 0.02). These findings are consistent with previous work on desktops^47–50^. To summarize, in this section, we replicated the key findings on oculomotor tasks such as prosaccade, smooth pursuit and visual search tasks using our smartphone eye tracker.

### Validation on natural images

We further validated our method by testing whether previous findings on eye movements for rich stimuli such as natural images, obtained from expensive desktop eye trackers with large displays could be replicated on small displays such as smartphones, using our method. Some well-known phenomena about gaze on natural images are that gaze is affected by (a) the task being performed (known since the classic eye tracking experiments by Yarbus in 1967^30^); (b) the saliency of objects in the scene^19,51,52^; and (c) tendency to fixate near the center of the scene^51,53^. To test whether our smartphone eye tracker can reproduce these findings, we collected data from 32 participants as they viewed natural images under two different task conditions: (1) free viewing and (2) visual search for a target (see “Methods”, study 3).

**Fig. 5:**
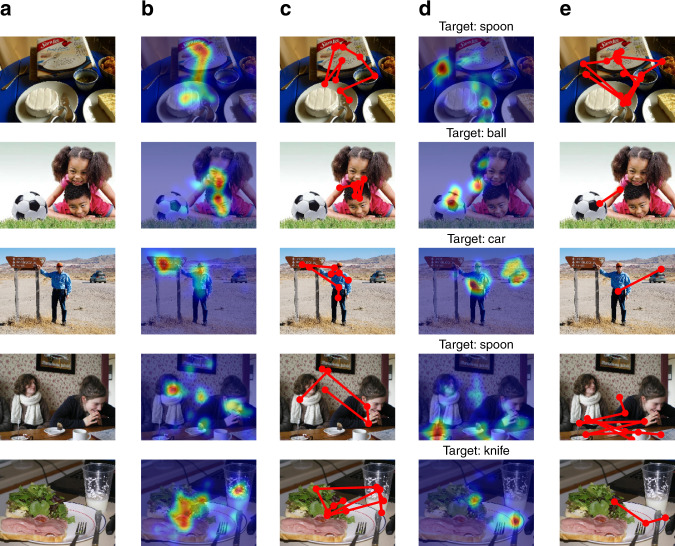
Gaze on natural images depends on the task being performed. The columns refer to: **a** Original image; **b** fixation heatmap during free viewing; **c** example scanpath from a single participant for free viewing; **d** fixation heatmap during visual search for a target object (specified in the title of each image); **e** example scanpath from a single participant for the visual search task.

As expected, gaze patterns were more dispersed during free viewing, and more focused toward the target object and its likely locations during visual search (see Fig. 5). For example, Fig. 5 third row shows that during free viewing, participants spent time looking at the person, and the sign he points to in the scene, while during visual search for a “car”, participants avoided the sign and instead fixated on the person and the car. Across all images, gaze entropy was found to be significantly higher for free viewing than for visual search (16.94 ± 0.03 vs. 16.39 ± 0.04, t(119) = 11.14, *p* = 10^−23^). Additional analysis of visual search performance showed that consistent with previous findings^54^, the total fixation duration to find the target decreased with the size of the target (*r* = −0.56, *p* = 10^−11^; *n* = 120 images), confirming that bigger targets are easier to find than smaller ones. Beyond size, we found that target saliency density has a significant effect on time to find the target (*r* = −0.30, *p* = 0.0011; *n* = 120 images), i.e., more salient targets are easier to find than less salient ones, consistent with previous literature^19^.

**Fig. 6:**
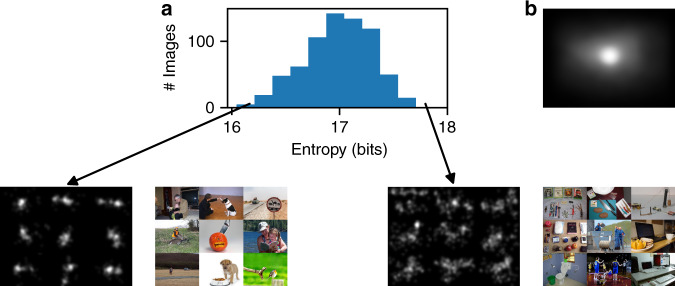
Gaze entropy and center bias during free viewing on phones. **a** Histogram of gaze entropy across all images for the free viewing task along with examples of low vs. high entropy images. **b** Averaging the fixations across all users and images reveals a center bias.

Second, we tested the existence of the central tendency during free viewing of natural images on smartphones. Figure 6a shows the gaze entropy across all images in this study. Examples of low gaze entropy are images containing one or two salient objects in the scene (e.g., a single person or animal in the scene), while the high entropy images contain multiple objects of interest (e.g., multiple people, indoor room with furniture). Similar findings were reported with specialized desktop eye trackers^51,52^. Averaging the fixations across all users and images from our smartphone eye tracker revealed a center bias (see Fig. 6b), consistent with previous literature on desktops^51,53^.

Finally, since saliency has been extensively studied using desktop eye trackers^19,51,52^, we directly compared the gaze patterns obtained from our smartphone eye tracker against those obtained from specialized desktop eye trackers such as Eyelink 1000 (using the OSIE dataset^52^). Note that this comparison places a high bar. Not only did the desktop setup with EyeLink 1000 involve specialized hardware with infrared light source and infrared cameras near the eye with high spatio-temporal resolution (up to 2000 Hz), but it also used highly controlled settings with chin rest (and dim lighting conditions), and displayed the image on a large screen (22*″*, 33 × 25^∘^ viewing angle). In contrast, our study setup used the smartphone’s existing selfie camera (RGB) in more natural settings (natural indoor lighting, no chin rest, just a stand for the phone) with images viewed on a small mobile screen (6*″*, median viewing angle of 12 × 9^∘^). Thus, the two setups differ in a number of ways (large-screen desktop vs. small-screen mobile, controlled settings, eye tracker cost, sampling rate).

**Fig. 7:**
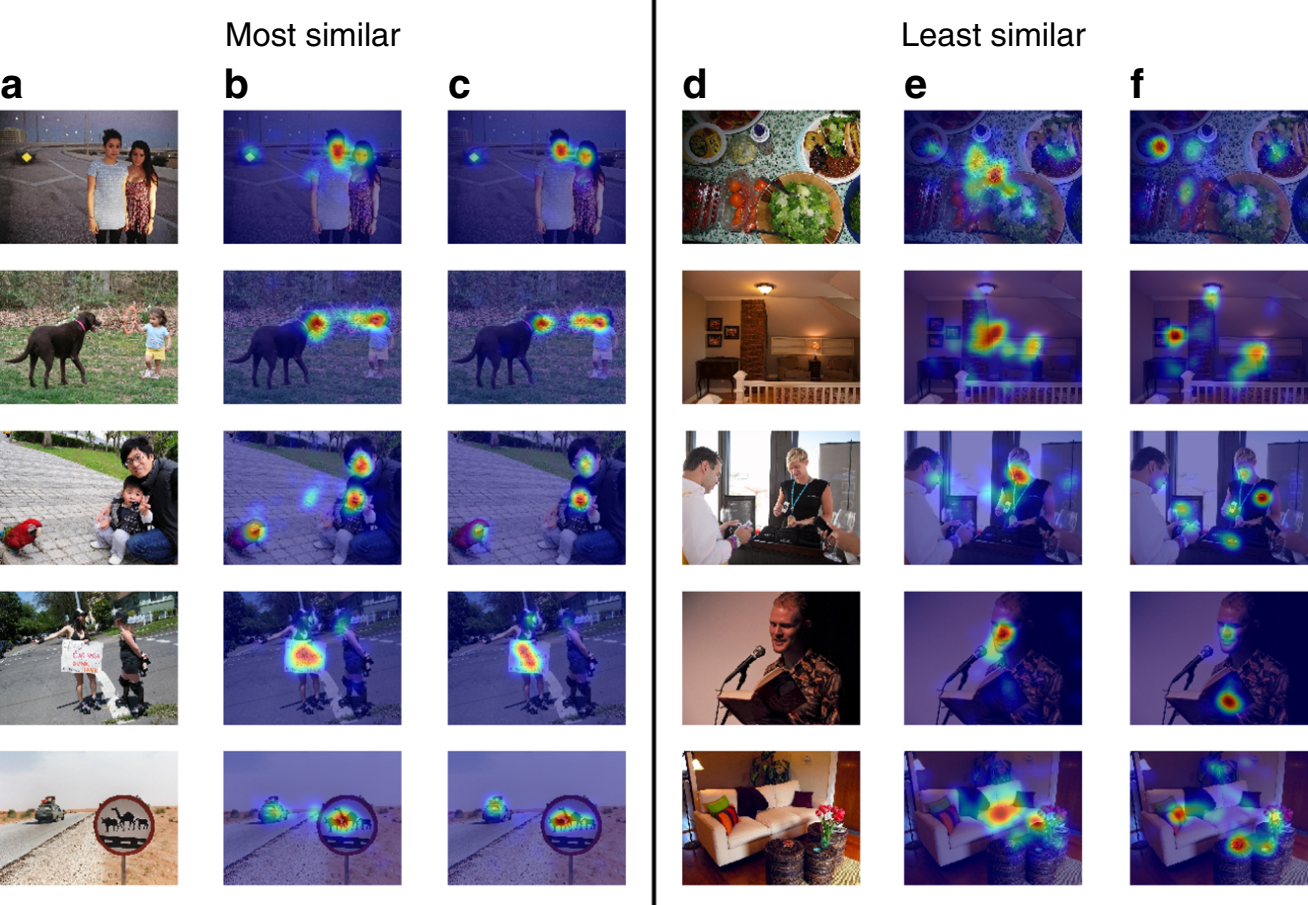
Comparison between mobile and desktop gaze for natural image viewing. The left hand side shows the most similar mobile vs. desktop heatmaps, while the right hand side shows the least similar heatmaps. Columns refer to: **a** and **d** original image; **b** and **e** mobile gaze heatmap with a blur width of 24 px; **c** and **f** desktop gaze heatmap with a blur width of 24 px (corresponding to 1^∘^ desktop viewing angle). See Supplementary Fig. 9 and Supplementary Table 1 for similar results with a larger blur width of 67 px (corresponding to 1^∘^ mobile viewing angle).

Despite these differences, we found that the gaze heatmaps from the two settings are qualitatively similar. Figure 7 shows the most similar and dissimilar heatmaps from desktop vs. mobile (similarity measured using Pearson’s correlation). Our smartphone eye tracker was able to detect similar gaze hotspots as the expensive desktop counterparts, with a key difference being that the mobile gaze heatmaps appear more blurred (see Supplementary Discussion for further analysis). The blur is due to a combination of the small size display on the mobile screen, and the lower accuracy/noise from the smartphone eye tracker (no chin rest, no infrared cameras near the eye). Apart from the blur, the gaze heatmaps from desktop and mobile are highly correlated both at the pixel level (*r* = 0.74) and object level (*r* = 0.90, see Table 1). This suggests that our smartphone eye tracker could be used to scale saliency analyses on mobile content, both for static images and dynamic content (as participants scroll and interact with the content, or watch videos).

**Table 1:** Correlations between mobile and desktop gaze.

	Corr(mobile, desktop)	Shuffled desktop correlation	Corr(desktop, centerBias)
Pixel-level correlation	0.74	0.11	0.26
Object-level correlation	0.90	0.59	0.76

Columns show Pearson’s correlation between the desktop and (1) mobile heatmap from our study; (2) desktop heatmap from a randomly selected image; (3) Gaussian centered at the image. Rows show the pixel- and object-level correlations.

### Testing on reading comprehension task

**Fig. 8:**
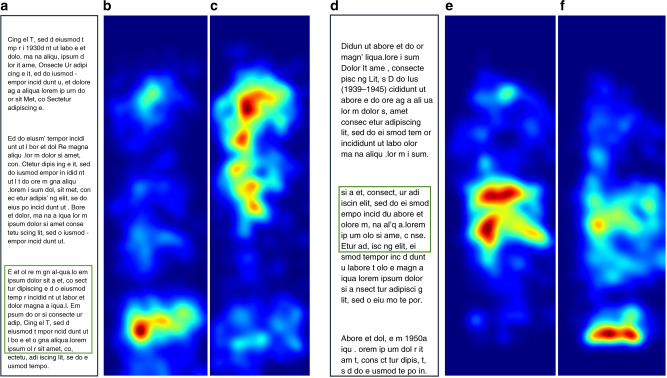
Different gaze patterns for factual vs. interpretive tasks. **a** Sample passage shown to the participant (actual text replaced with dummy for copyright reasons). Green bounding box highlights the relevant excerpt for the factual task (box shown for visualization purposes only, participants did not see this). **b** Population-level gaze heatmap for the factual task, for the passage shown in (**a**). **c** Heatmap for the interpretive task for the passage shown in (**a**). **d**–**f** Similar to (**a**–**c**) except that the factual task appeared after the interpretive task. In both examples, gaze was more dispersed across the passage for interpretive than factual tasks.

Beyond research validation on oculomotor tasks and natural images, we tested whether our smartphone eye tracker could help detect reading comprehension difficulty, as participants naturally scrolled and read passages on the phone. Seventeen participants read SAT-like passages on the phone (with scroll interactions), and answered two multiple choice questions (see “Methods”, study 4). One of the questions was factual and could be answered by finding the relevant excerpt within the passage. The other question required interpreting the passage in more detail—we call this the “interpretive” task. As expected, we found that the gaze patterns are different for factual vs. interpretive tasks. Gaze patterns were more focused on specific parts of the passage for factual tasks, and more dispersed across the passage for interpretive tasks (see Fig. 8). Across all users and tasks, gaze entropy was found to be higher for the interpretive tasks than the factual tasks (8.14 ± 0.16 vs. 7.71 ± 0.15; t(114) = 1.97, *p* = 0.05).

**Fig. 9:**
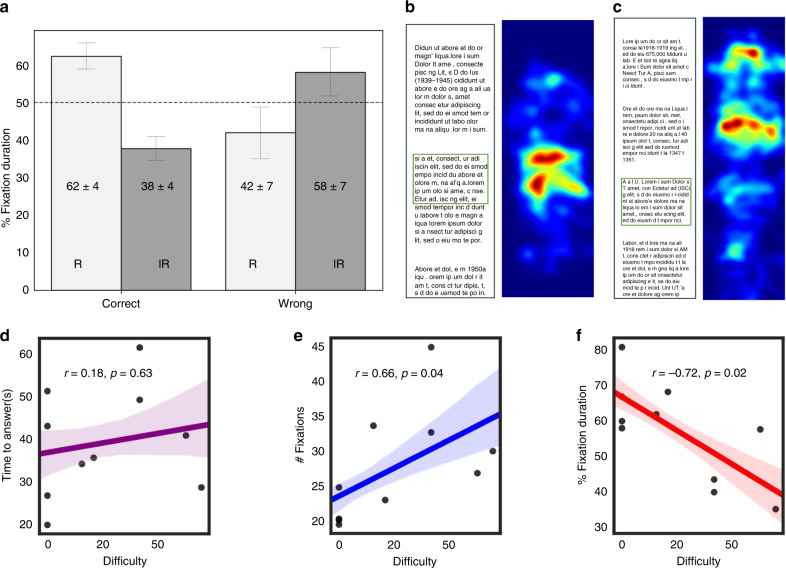
Effect of reading comprehension difficulty on gaze for factual tasks. **a** Barplot shows % fixation duration on the relevant portion of the passage (normalized by height) when participants answered the factual question correctly vs. not. Error bars denote the mean ± s.e.m. (*n* = 53, 13 tasks for correct vs. wrong responses). **b** Example of fixation heatmap for easy factual task; **c** difficult factual task. **d**–**f** Scatterplots showing different metrics as a function of task difficulty. **d** Time to answer the question in seconds (includes time spent reading the question and the passage); **e** number of fixations on the passage; **f** percentage time on relevant region, computed as the % total fixation duration on the relevant portion of the passage (normalized by height). Statistical correlation reported is the Spearman’s rank correlation coefficient (*n* = 10 tasks); two-tailed one sample *t*-test. The confidence band represents the bootstrapped 68% confidence interval.

Within factual tasks, we examined if there are differences in gaze patterns when participants answered the question correctly vs. not. We hypothesized that gaze should be focused on the relevant excerpt in the passage for participants that answered correctly, and gaze should be more dispersed or focused on other parts of the passage for incorrect answers. Figure 9a shows that participants spent significantly more time fixating within the relevant passage regions than irrelevant ones when they answered correctly (62.29 ± 3.63% time on relevant vs. 37.7 ± 3.63% on irrelevant; t(52) = 3.38, *p* = 0.001). This trend was inverted for wrong answers, though not significant (41.97 ± 6.99% on relevant vs. 58.03 ± 6.99% on irrelevant; t(12) = −1.15, *p* = 0.27).

Next, we examined the effect of task-level difficulty on gaze and time-to-answer. We quantified task difficulty as the %incorrect answers per task (see Supplementary Figs. 6–7 for additional measures of task difficulty that take time and accuracy into account). Figure 9b–f shows example gaze heatmaps for easy vs. difficult tasks, and the corresponding scatterplots of various metrics as a function of task difficulty. As expected, time to answer increased with task difficulty, though not significantly (Spearman’s rank correlation *r* = 0.176, *p* = 0.63). The number of eye fixations on the passage increased with task difficulty (*r* = 0.67, *p* = 0.04). A closer look showed that the best predictor was fraction of gaze time spent on the relevant excerpt (normalized by height), which was strongly negatively correlated with task difficulty (*r* = −0.72, *p* = 0.02). In other words, as task difficulty increased, participants spent more time looking at the irrelevant excerpts in the passage before finding the relevant excerpt that contained the answer. These results show that smartphone-based gaze can help detect reading comprehension difficulty.

## Discussion

We overcome the high cost and lack of scalability of specialized eye trackers by demonstrating accurate smartphone-based eye tracking without any additional hardware. Leveraging machine learning with smartphone’s front-facing camera feed as input, our model achieves 0.46 cm error on phone screen (0.6–1^∘^ viewing angle) using under 30 s of calibration data per user. This accuracy is comparable to state-of-the-art mobile eye trackers like Tobii glasses, that are at least 100× more expensive (~$30K vs.  ~$150 for ours). We validate our methodology by reproducing the key findings from previous eye movement research on ocolomotor tasks (including prosaccade, smooth pursuit, visual search) and saliency analyses for natural images obtained using bulky, expensive desktop eye trackers with chin rest and 3× larger displays. Beyond research validation, we demonstrate that smartphone gaze can help detect reading comprehension difficulty as participants scroll and read passages on the phone.

Unlike the high-end eye trackers used in vision research so far, our method does not require any specialized hardware, is inexpensive, and works with existing front-facing camera of smartphones. This offers the potential to scale eye tracking studies along three axes: (1) across new and diverse set of applications that previously did not consider eye tracking due to the high costs and complexity; (2) across broader and diverse population, especially in the developing world as smartphone penetration is rapidly increasing; (3) across larger number of participants for a given study as our method can be leveraged to scale eye tracking studies by orders-of-magnitude to several thousands of participants in remote settings.

Our study has some limitations. We brought participants into the lab, and used a fixed device stand to avoid strain from holding the device for 45 min, and to avoid large changes in headpose. Future work will explore more natural settings like hand-holding the device in remote settings. The temporal resolution of our smartphone-based eye tracker depends on the phone being used (i.e., selfie camera specs). In this paper, we used Pixel 2 XL phones whose temporal resolution is low (30 Hz) compared to 50 Hz for mobile Tobii glasses or 1000–2000 Hz for the desktop Eyelink 1000. This limits precise measurements of saccade latencies, velocity, and fixation duration. Although high temporal resolution is not critical for many eye tracking tasks, as smartphone cameras continue to improve in temporal resolution (e.g., the slow motion mode on recent phones allows up to 240 Hz), our results will automatically improve, enabling more precise eye tracking measurements at a few millisecond resolution.

While this paper focused on Pixel 2 XL smartphones, our methodology can be used across devices (see Supplementary Fig. 3). We found that smartphone eye tracking works well with the following settings: frontal headpose (similar to desktop eye tracking studies); distance to the phone adjusted such that the face covers most of the front-facing camera frame; good indoor lighting conditions (avoid dark rooms, bright lights, windows or reflective screens in the background); and participants with normal vision, without glasses (to avoid reflection from the glasses). As seen in Supplementary Fig. 3c–f, some of the main failure cases include extreme headpose (tilt/pan/roll), when participants look down (eyes appear partially closed), or when they hold the phone far away (eye appears small). Future work includes improving model robustness and performance across different head poses, distance, devices as well as across demographics to help democratize eye tracking.

One area that could benefit tremendously from smartphone eye tracking is gaze-based interaction for accessibility^27,55^. People with conditions such as Amyotrophic lateral sclerosis (ALS), locked-in syndrome, stroke, and multiple sclerosis have impaired speech and motor ability which limits their ability to touch and interact with the phone/tablet. Smartphone eye tracking could provide a powerful way to transform their lives by using gaze for interaction. This requires gaze to be estimated on-device and real-time. There may be additional challenges due to head shaking or tremors in certain conditions. Nevertheless, the potential to scale eye tracking for accessibility is exciting.

Another area that could benefit from smartphone eye tracking is screening and monitoring of health conditions. Eye movement behavior is known to be abnormal for certain health conditions like autism spectrum disorder (ASD)^28^, dyslexia^56^, concussion^43,57^, and more. For example, patients with ASD tend to avoid looking at the eyes and instead preferentially fixate on the nose or mouth of faces, compared to healthy controls^28^. Patients with concussion or mild traumatic brain injuries have difficulty performing a smooth pursuit task, such as tracking an object moving in a circle or box^43,57^. By scaling these studies to the population level via smartphone eye tracking, we could enable gaze as a scalable, digital phenotype for screening or monitoring progression of health conditions, which could reduce healthcare spending by providing timely, early interventions and saving the need for costly doctor visits, especially for countries with limited access to healthcare services.

While smartphone eye tracking could enable a wide array of useful applications, it is important to be mindful of the correct use of this technology, requesting explicit approval and fully informed consent from users for the specific application at hand. In this paper, all the data was collected in lab settings for research purposes with users’ explicit consent. In addition, users were allowed to opt out of the study at any point and request their data to be deleted, without affecting their compensation for participating in the study. The data in the current study was processed offline, by moving the data to our servers, where they were encrypted and stored (with restricted access) for data analysis. We plan to mitigate the privacy concerns further in future work by running the model entirely on device.

In conclusion, our demonstration of accurate ML powered smartphone eye tracking with accuracy comparable to state-of-the-art specialized mobile eye trackers offers the potential to scale eye tracking studies from few ten participants in the lab to thousands of participants in remote settings. This unlocks unique opportunities across a number of areas including basic vision research, reading and language understanding, usability research; in addition, it enables applications for societal good such as gaze-based interaction for accessibility, detecting comprehension difficulty in education, and smartphone-based screening/monitoring tools for healthcare.

## Methods

### Model

We used a multi-layer feed-forward convolutional neural network (ConvNet) similar to previous work^37,58^. The face features for each image (face bounding box, six landmarks) were extracted using a face detector built on MobileNets^59^ with the SSD detector^60^. This base model was trained on the MIT GazeCapture dataset^37^. Eye regions were cropped based on the eye corner landmarks, scaled to 128 × 128 × 3 pixels, and fed through two identical ConvNet towers, one for each eye. Each tower consisted of three convolutional layers with kernel sizes 7 × 7, 5 × 5, and 3 × 3, for the first, second, and third layer. The three layers had 32, 64, and 128 output channels, respectively. The first two kernels were applied with a stride of 2, and the final one with a stride of 1. Rectified linear units (ReLUs) were used as nonlinearities. Each convolutional layer was followed by an average pooling layer of size 2 × 2. The left eye crop was flipped horizontally to allow shared weights between the two towers to simplify training. Inner and outer eye corner landmarks (4 × 2 floating point numbers) were sent through three successive fully connected layers, and combined with the output of the two towers by two additionally fully connected layers. The number of hidden units for layers 1–5 were 128, 16, 16, 8, and 4, respectively. A regression head outputs two numbers for the *x*- and *y*-location of gaze on the phone screen. Additional details available in the Supplementary.

Model accuracy was improved by adding fine-tuning and per-participant personalization. Calibration data (see next paragraph) was recorded over a period of  ~30 s, resulting in  ~1000 input/target pairs. The base model described above was fine-tuned with the calibration data from all users. During fine-tuning, all the layer weights of the pre-trained base model were allowed to be updated until the model converged. Subsequently, feature images were processed by the gaze predictor, and a lightweight regression model was fitted to the output from the fine-tuned model’s penultimate ReLU layer to produce *x* and *y* screen coordinates (or gaze estimates) that minimize the deviation to the targets (ground truth gaze). We chose support vector regression (SVR) for the lightweight model. During inference, the pre-trained base model and the regression model were applied in sequence to an image to generate the final, personalized gaze estimate. Model accuracy was evaluated across all participants by computing the error in cm between stimulus locations from the calibration tasks (ground truth) and the estimated gaze locations.

For calibration tasks, participants were asked to fixate on a green circular stimulus that appeared on a black screen. For dot calibration, visibility of the stimulus was improved by having it pulsate in size between 18 and 50 density-independent pixels (dp), once every 300 ms. For zig-zag calibration, the dot moved slowly from top-left to the bottom-right in a zig-zag fashion for 60 s. Images from the front-facing camera were recorded at 30 Hz and timestamps synchronized with the marker location.

Model accuracy for the personalized model was evaluated across all participants of the user studies, for a combined total of over 100 participants. Stimulus locations from the calibration tasks were compared to estimated gaze locations to obtain the error in cm and yielded an average model error of 0.46 cm on-device screen (at a viewing distance ranging from 25 to 40 cm) across participants.

### Data collection and analysis

External participants were recruited from a pool of user study volunteers who signed up through the Google User Experience Research portal^61^. Each participant provided their explicit and informed consent to data collection by reading and signing a study-specific participant agreement that informed them about collecting the front-facing camera feed for research analyses purposes, and the potential risks involved in performing gaze tasks for several minutes (e.g., eye strain, fatigue). Participants received monetary compensation for their time even if they did not complete the tasks, and retained the option to have their data deleted at any time. Studies were designed to be  <45 min in length and were conducted in lab settings. A facilitator provided instructions and was present at all times. All studies and data collection were consistent with Google Privacy, Legal and Ethics policy. The authors affirm that human research participants provided informed consent for publication of the images in Fig. 2.

Data was collected with a custom Android app. The app served two main purposes: (1) to display the stimulus along with task instructions on screen; (2) capture and store the front-facing camera feed, as well as user click/scroll/touch interactions on the screen. The sequence and content of screens were study-specific and predetermined. All studies were conducted with the phone in portrait mode. While the model is light enough to be run on-device for real-time readouts, to allow a flexible comparison of multiple models on the same input (facial images), the current implementation used the phone primarily for stimulus display and recording the front-facing camera feed, while the processing and readouts were performed offline.

Depending on the task, the stimulus appeared at random locations on the screen (dot calibration), or moved smoothly across the screen in a circular, rectangular, or zig-zag pattern from the upper left to lower right corner (smooth pursuit). For all studies, we extracted raw gaze images and event logs from the study phones to obtain personalized gaze estimates and participants with error >1 cm on a hold-out calibration dataset were discarded.

Eye movements were classified according to the velocity of eye movements, with saccades and fixations determined by a velocity threshold (22^∘^ s^−1^), similar to the method described in prior work^62^.

### Study 1: Comparison with specialized mobile eye trackers

This study was conducted with 30 participants from the San Francisco Bay Area, aged 18 and above of whom 65% identified as male, all others as female.

This study was performed with Tobii Glasses Pro 2 and Pixel 2 XL phone. The tests were identical to the dot and zig-zag calibration tasks. For the dot task, a sequence of 41 dots was shown over 1 min, while the zig-zag task lasted 60 s. The tasks were performed four times: with Tobii glasses vs. without them, and with the phone handheld vs. mounted on a device stand.

The Tobii Pro Lab software used with Tobii Glasses required a one-point calibration at the beginning. The software then mapped the estimated gaze direction to objects in the scene captured by the device’s world-facing camera. In practice, manual intervention was often required to achieve correct mapping of the phone’s screen. To improve robustness of the method, we added fiducials in the form of AprilTags^63^ to the four corners of the display. We imported a snapshot of the resulting background into the Tobii software for automapping. Participants for whom automapping completely failed (*n* = 9) were discarded. In addition, participants with error  >1 cm (*n* = 4) using Tobii were dropped for the comparison (see Supplementary Fig. 3 for results including these participants). Post data cleaning, the dataset contained 26 participants for our model, and 13 participants for the comparison between Tobii and our model.

To allow the eye to fixate on a stimulus (discounting saccade latency), initial 800 ms of frames captured after the onset of each stimulus were discarded. Personalization was performed based on the zig-zag calibration task. For each dot stimulus, the median gaze estimate across all frames for that stimulus was computed. For Tobii, the automapped gaze estimates on the phone screen were recorded. Gaze estimates that ended up outside the screen area were snapped to the nearest valid screen location. Estimates for each dot location were also median filtered.

The Tobii Pro Glasses captured images at a rate of 50 Hz, while the Pixel 2 XL phone recorded images at 30 Hz. We rendered the two comparable by providing a single aggregate estimate for each of the 41 dots, and by comparing Euclidean distance between the stimulus location (ground truth) and estimated gaze (from Tobii vs. our model).

### Study 2: Oculomotor tasks

This study was conducted with 30 participants from San Francisco Bay Area, aged 18–55, with genders equally split between male and female. Post data cleaning, the dataset contained 22 participants for subsequent analysis.

The oculomotor test consisted of prosaccade, smooth pursuit, and visual search tasks. Tasks were divided into six blocks 3–5 min in length, with 1 min of rest between blocks. Participants completed a dot calibration task at the beginning of the study and once per block.

For each repetition of the prosaccade test, participants were asked to first focus on a yellow marker in the shape of a cross (168 pixels in size) at the center of the screen. The marker was then replaced by a green circular marker, the visual stimulus. The stimulus’s location was uniformly sampled across the entire horizontal and vertical screen area. To increase visibility, both the center marker and stimulus pulsated in size (between 60 and 168 pixels) thrice, for a total duration of 1000 ms. Participants were instructed to move their gaze toward the stimulus as soon as it appeared. Participants performed ten trials per sub-block, and three sub-blocks of prosaccade tasks.

For the smooth pursuit test, participants were presented with a green circular stimulus similar to that of the prosaccade test, but this time non-pulsating and moving across the screen along a predetermined path. The path was circular and clockwise around the screen center, starting and ending on the right-most point of the circle close to the right edge of the screen, at an eccentricity of  ~7^∘^. Similarly for smooth pursuit box task. Participants performed a total of three trials each of smooth pursuit circle and box tasks, with one such trial randomly chosen per 5 min block of the study.

The visual search task was performed in two variants, one for intensity and one for object orientation. For the former, participants were presented with a set of circles on an otherwise blank screen. One of the circles was displayed in the same color, but with a different color intensity from the rest. For the latter, a set of shapes was shown, one in a different orientation from the rest. In both cases, participants were instructed to tap on the screen location of the diverging object with their finger. See the Supplementary Discussion for details.

For the prosaccade and smooth pursuit tasks, estimated gaze locations were time-synchronized with the stimulus location (ground truth) and used for subsequent data analysis (e.g., computing error, saccade latency). For each visual search task, we computed the number of fixations and the total fixation duration taken to find the target in the display.

### Study 3: Saliency

This study was conducted with 37 participants from New York City. Post data cleaning, the dataset contained 32 participants for subsequent analysis.

This study consisted of three tasks: calibration, free viewing of images, and visual search of predetermined objects within images. Images were shown sequentially in multiple blocks, with each block book-ended by a calibration task to improve robustness to drifts in gaze over time. To prevent fatigue, blocks were separated by 60-s breaks. Phones were mounted on a stand in front of the participants. For calibration, participants were asked to complete rectangular smooth pursuit, zig-zag smooth pursuit, and random dot tasks.

Seven hundred images of natural indoor and outdoor scenes from the OSIE dataset^64^ were used. Each participant was shown a random selection of 350 images for 3 s each, with images separated by 1 s of a blank screen. The final dataset had an average 16 participants per image.

Hundred and twenty images were selected from the labeled OSIE dataset for the visual search task. For each trial, participants saw the label of the target object (one of ‘person’, ‘dog’, ‘laptop’, ‘phone’, ‘car’, ‘ball’, ‘spoon’, -‘knife’, ‘ship’, and ‘hat’), followed by an image containing that object. They were asked to find the object in the image and tap its location on screen. Ten blocks of six images were presented to each user.

The raw gaze was then smoothed with a bilateral filter (with a filter width of 100 ms and 200 pixels). Fixations were extracted with a simple velocity filter using a velocity threshold of 22^∘^ s^−1^ and a min fixation time of 100 ms. We obtained fixation maps by rounding gaze locations to the nearest pixel. Saliency maps were created by applying a Gaussian filter of size 24 pixels to match the filter sized used by the OSIE study.

### Study 4: Reading comprehension

Twenty-three participants from San Francisco Bay Area, aged 18–54 took part in this study. Seventy-eight percent of participants self-identified as native speakers, the rest reported good English fluency. Seventy-four percent identified as male, all others as female. All participants reported at least high-school knowledge of computer science. Post data cleaning, the dataset contained 17 participants for subsequent analysis.

The education study consisted of calibration and ten reading comprehension tasks, of which five tested general English reading (SAT-like passages), and five tested reading computer science passages, that involved documentation explaining technical concepts, interspersed with code snippets. Each task was followed by two reading comprehension questions, one factual, and one interpretive. The answer to a factual question could be found directly in the passage, while the interpretive question required inference from the passage to answer correctly. Between reading tasks, participants completed a dot calibration task followed by zig-zag smooth pursuit.

Personalized gaze estimates were obtained using the calibration tasks (similar to study 1). The reading tasks involved scrolling. To account for changing screen content, viewport information was time-synchronized with gaze estimates to compute gaze on page-level coordinates. This allowed subsequent analysis including generating gaze heatmaps across entire passages by aggregating gaze estimates from multiple participants.

### Ethical considerations

Google’s AI Principles were a key consideration in the release of this publication. The primary purpose of this technology is to drive societal benefit by increasing the ubiquity of eye gaze technology and drive breakthroughs in vision-based applications to make products more accessible and enable advances in healthcare. For those choosing to carry on this research or adopt its findings for novel applications, consideration should be given to ensuring there is adequate representation from diverse populations in ML models and that there are sufficient methods of obtaining informed consent by users.

### Reporting summary

Further information on research design is available in the Nature Research Reporting Summary linked to this article.

## Supplementary information

Supplementary Information

Reporting Summary

## Data Availability

Gaze estimates (inferred *x*- and *y*-locations on screen) for the studies are available from the corresponding author [V.N.] upon reasonable request. To protect study participant privacy and consent, the captured full face image data are not publicly available. A reporting summary for this article is available as a Supplementary Information file. Source data are provided with this paper.

## Source data

Source Data

## Code availability

We made use of the open source machine learning frameworks TensorFlow (https://github.com/tensorflow/tensorflow) and scikit-learn (https://scikit-learn.org) to conduct our experiments. Since many aspects of the experimental system such as data generation and model training have a large number of dependencies on internal tooling, infrastructure and hardware, we are unable to publicly release this code. However, all the experiments and implementation details (including data augmentation, model architecture, and hyperparameters) are available in the “Methods” and Supplementary Information, to allow for independent replication. In addition, a binary of the trained neural network of the base model is available for academic purposes upon reasonable request from the corresponding author [V.N.].
