# Supplementary material for: Accelerating eye movement research via accurate and affordable smartphone eye tracking
Source: Nat Commun. 2020 Sep 11;11:4553. doi: 10.1038/s41467-020-18360-5 (PMC7486382; doi:10.1038/s41467-020-18360-5)
Supplement: Supplementary file 1 — Supplementary Information [file 41467_2020_18360_MOESM1_ESM.pdf]

Supplementary Information

Accelerating Eye Movement Research via Accurate and Affordable Smartphone Eye Tracking

Nachiappan Valliappan[1], Na Dai[1], Ethan Steinberg[†1], Junfeng He[1], Kantwon Rogers[‡1], Venky Ramachandran[1], Pingmei Xu[1], Mina Shojaeizadeh[1], Li Guo[§1], Kai Kohlhoff[1], and Vidhya Navalpakkam[*1]

[1]Google Research, Mountain View, CA, USA

July 2020

[†]Present address: Stanford University, Stanford, CA, USA
[‡]Present address: Georgia Institute of Technology, Atlanta, GA, USA
[§]Present address: Johns Hopkins University, Baltimore, MD, USA
[*]Corresponding author: vidhyan@google.com

Supplementary Figures

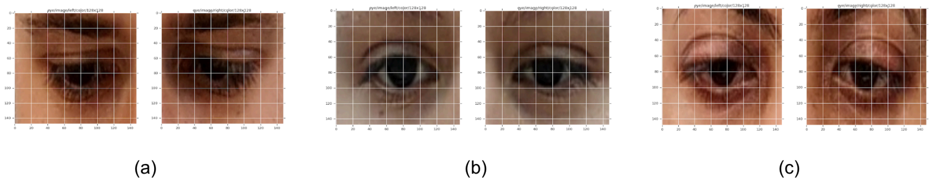

Supplementary Figure 1: Sample eye crops from Ps when they looked at the bottom of the screen. **a** P1's eyes appeared partially closed, resulting in higher errors in gaze estimation. **b** P2 did not seem to be performing the task correctly. **c** P3 had lower error since the eyes were still clearly visible.

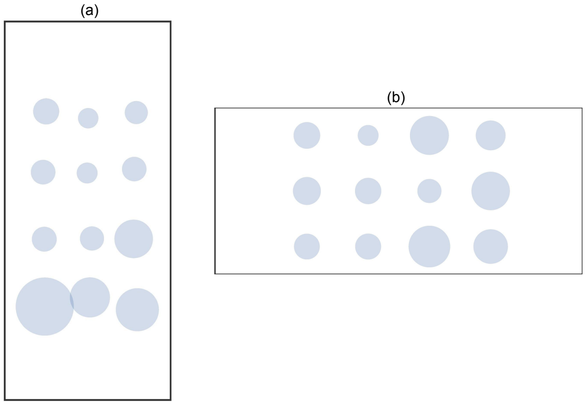

Supplementary Figure 2: Error as a function of location on screen for: **a** Tobii glasses in study 1, where the phone is held in portrait mode on the device stand; **b** Our method in study 1, where the phone is held in landscape mode on the device stand. The radius of the circle indicates average model error at that screen location.

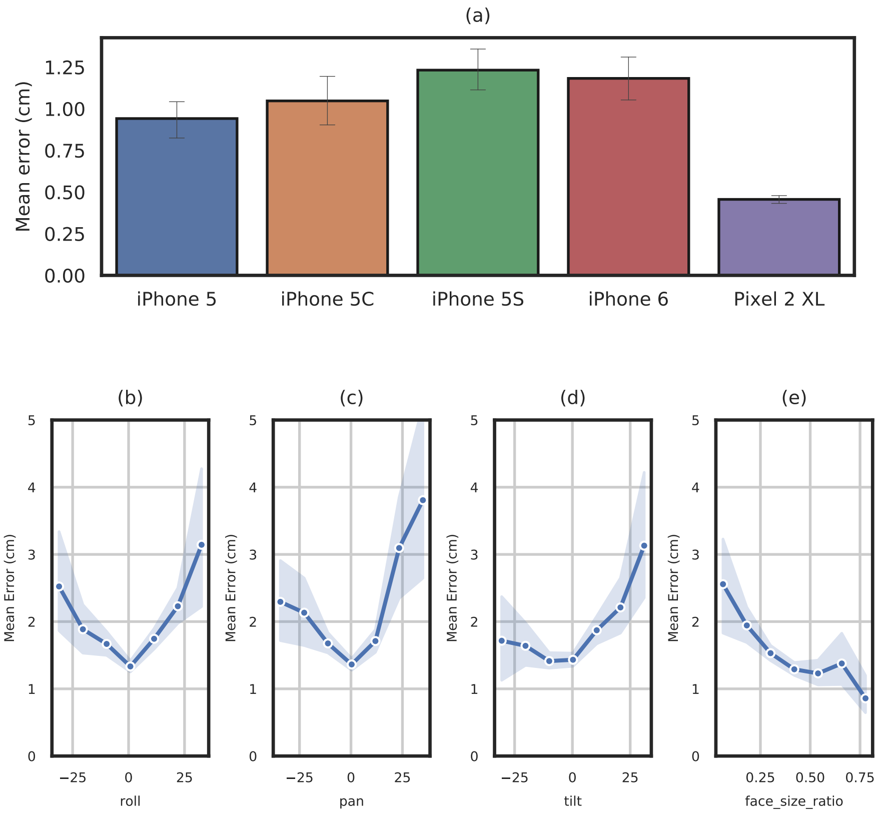

Supplementary Figure 3: **a** Accuracy after personalization across devices (n = {6,5,7,9,26} participants for {iPhone 5, iPhone 5C, iPhone 5S, iPhone 6, Pixel 2 XL} devices respectively). **b-e** Breakdown of accuracy by headpose (in degrees relative to centered) and distance (face size relative to screen size) for the GazeCapture dataset which had more natural diversity in viewing conditions. Error bars represent the mean ± s.e.m. for all the subfigures.

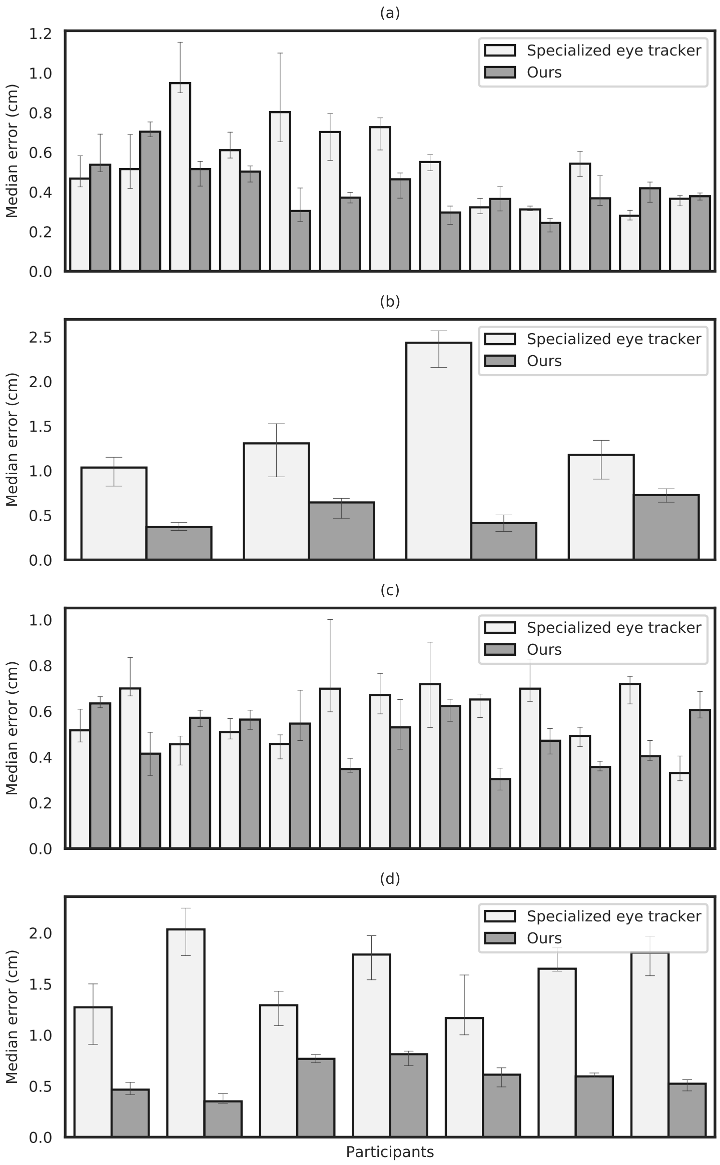

Supplementary Figure 4: Error distribution across users for Tobii glasses vs. our smartphone eye tracker. **a** Fixed device stand setting. **b** 4 Ps that were removed from the analysis due to higher error on Tobii. **c** Hand-held setting. **d** 7 Ps that were removed from the hand-held setting due to high Tobii errors. Error bars represent the mean ± s.e.m. (n = 41 independent calibration dot locations).

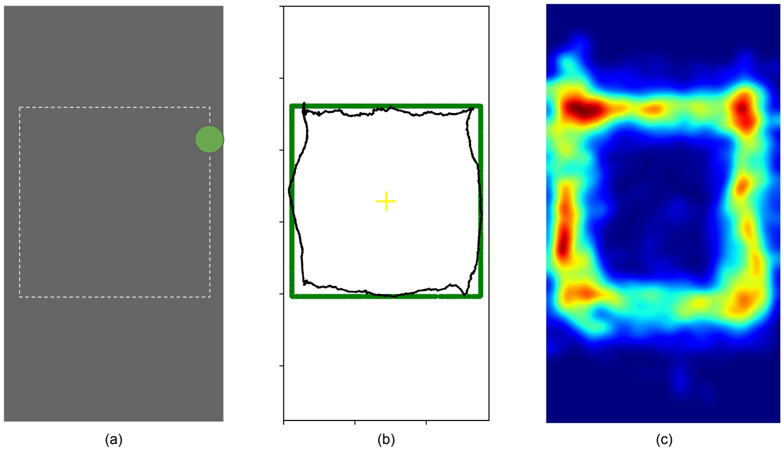

Supplementary Figure 5: Smooth pursuit task (box). **a** Participants were asked to look at the green dot as it moved along a box. **b** Sample scanpath from smooth pursuit task (box). **c** Population level heatmap from all users and trials for this task.

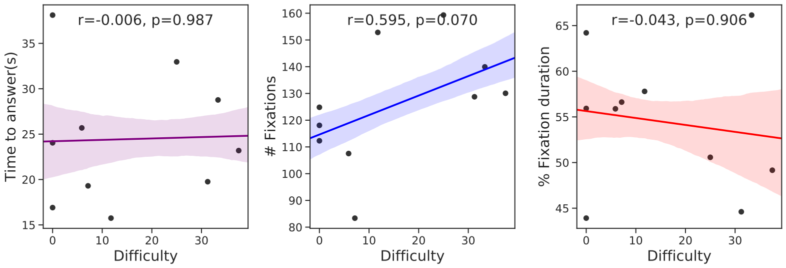

Supplementary Figure 6: Correlation of metrics vs. difficulty upon including the time spent on initial passage reading in addition to the "factual" time segment. Statistical correlation reported is the Spearman's rank correlation coefficient (n = 10 tasks); two-tailed one sample t-test. The confidence band represents the bootstrapped 68% confidence interval.

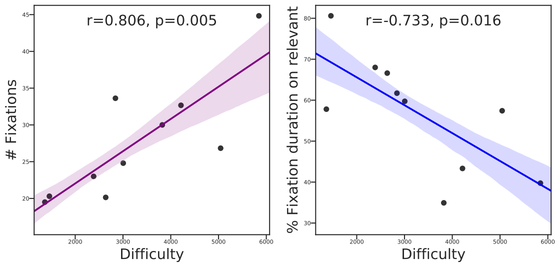

Supplementary Figure 7: Correlation between fixation metrics and a new measure of difficulty defined as the ratio of time to answer the task (s) over the accuracy. Statistical correlation reported is the Spearman's rank correlation coefficient ($n = 10$ tasks); two-tailed one sample t-test. The confidence band represents the bootstrapped 68% confidence interval.

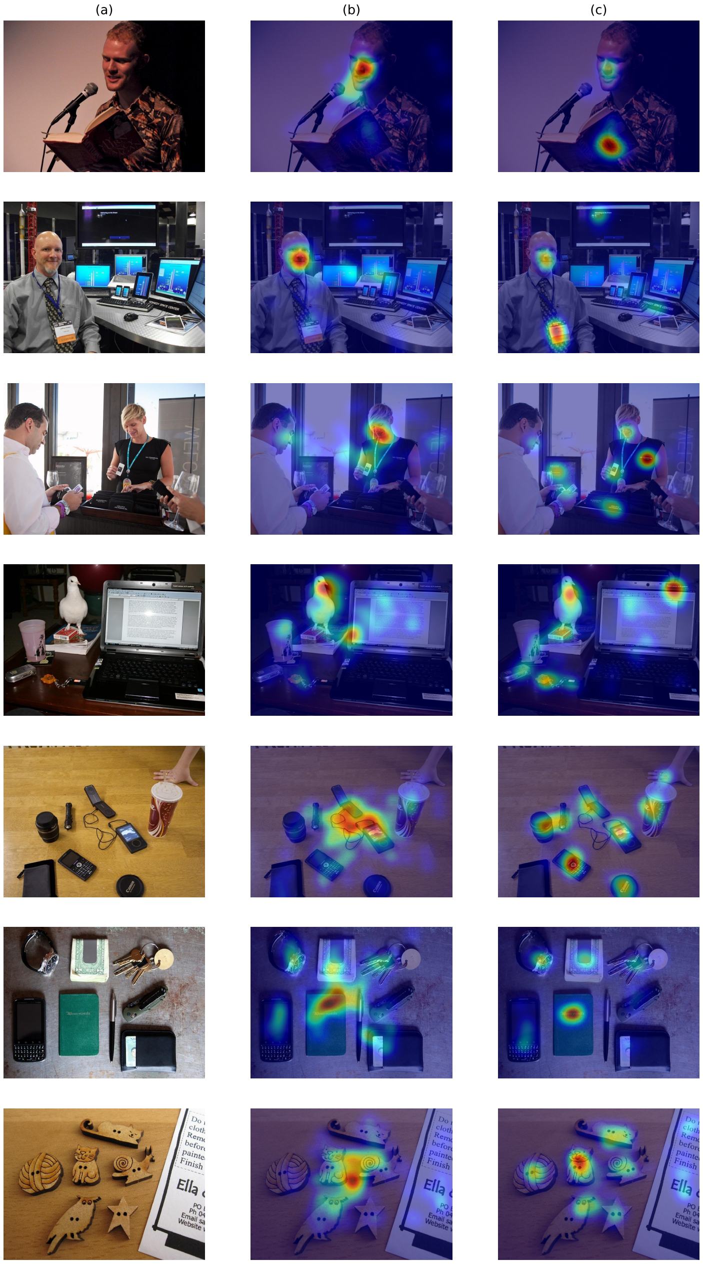

Supplementary Figure 8: Examples of least similar images between mobile and desktop gaze for natural image viewing. Columns refer to: **a** original image; **b** mobile gaze heatmap with a blur width of 24px; **c** desktop gaze heatmap with a blur width of 24px (corresponding to 1° desktop viewing angle). See the text for explanation of the differences.

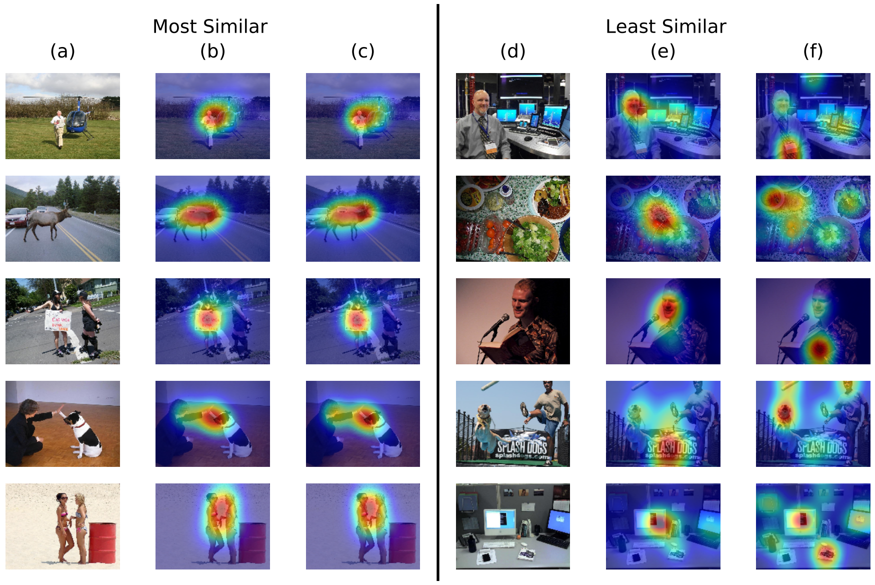

Supplementary Figure 9: Comparison between mobile and desktop gaze for natural image viewing. The left hand side shows the most similar desktop vs. mobile heatmaps, while the right hand side shows the least similar heatmaps. Columns refer to: **a & d** original image; **b & e** mobile gaze heatmap with a blur width of 67px; **c & f** desktop gaze heatmap with a blur width of 67px (corresponding to 1° mobile viewing angle).

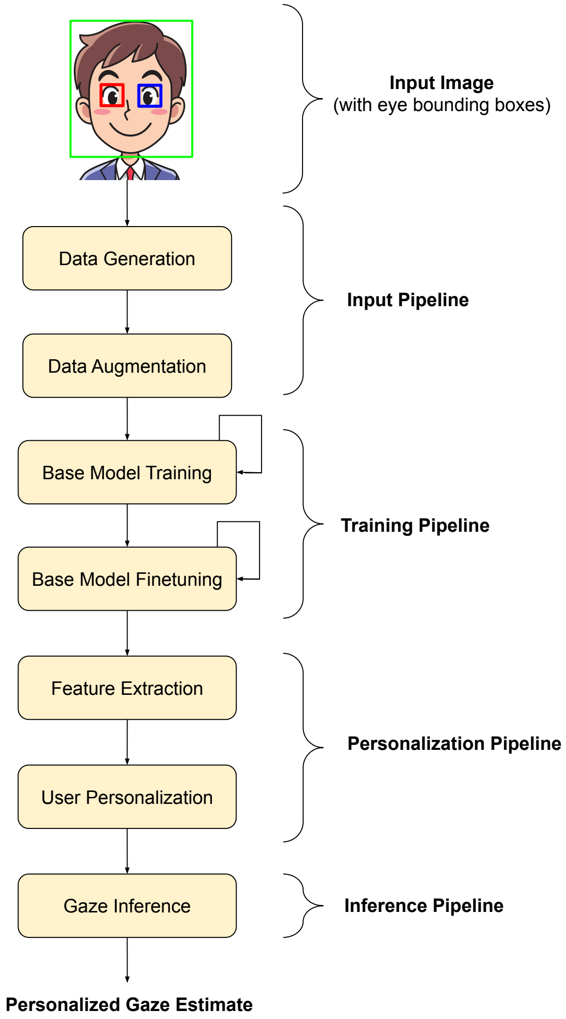

Supplementary Figure 10: Flow chart of the personalized gaze estimation system.

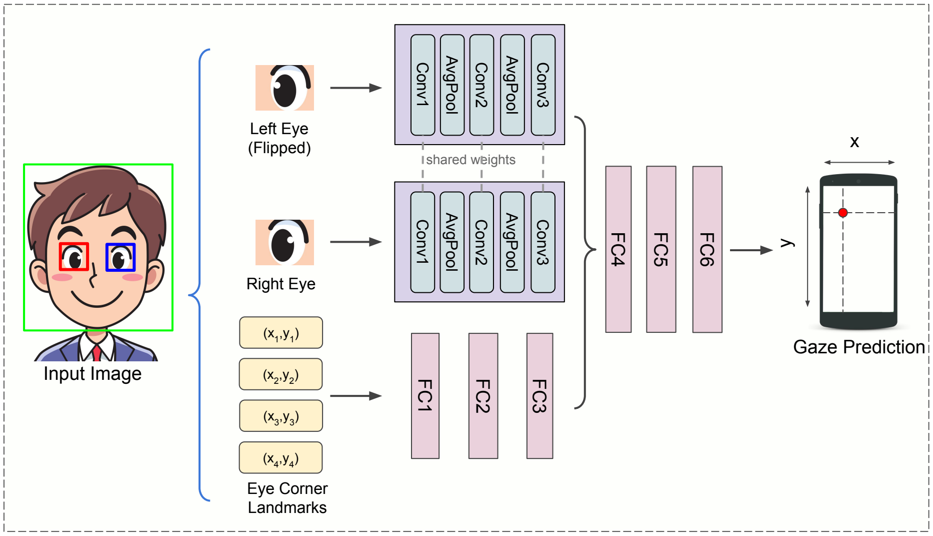

Supplementary Figure 11: Base model architecture. We use the following sizes for the convolutional layers: CONV1(7 x 7 x 32; stride = 2), CONV2(5 x 5 x 64; stride = 2), CONV3(3 x 3 x 128; stride = 1). Each convolutional layer was followed by an average pooling layer of size 2 x 2. The fully connected layers have the following sizes: FC1(128), FC2(16), FC3(16), FC4(8), FC5(4), FC6(2). Rectified Linear Units (ReLUs) were used as nonlinearities for all layers except the final FC6 output layer which has no activation.

Supplementary Tables

	Corr(desktop, mobile)	Shuffled desktop correlation	Corr(desktop, centerBias)
Pixel level correlation	0.874	0.291	0.459
Object level correlation	0.954	0.765	0.880

Supplementary Table 1: Correlations between mobile and desktop gaze.[1]

Supplementary Methods

Accuracy: Accuracy on screen was found to be lower at the bottom of the screen than top. A one-way between subjects ANOVA was conducted to compare the effect of row/column location on screen on gaze estimation error for the device stand condition. There was a significant effect of row/column on error

[1]Columns show the Pearson correlation between the desktop heatmap from OSIE dataset and 1) mobile heatmap from our study; 2) desktop heatmap from a randomly selected image; 3) Gaussian centered at the image. Rows show the pixel-level and object-level correlations.

for both row: $F(3, 1044) = 40.76$, $p = 6.58 \times 10^{-25}$ and column: $F(2, 1045) = 9.42$, $p = 9 \times 10^{-5}$. Similar results were obtained for the handheld condition.

Supplementary Figure 1 shows sample eye crops from three random Ps (say P1, P2 and P3) who were looking near the bottom middle right portion of the screen. Both P1 and P2 have > 1.2cm gaze estimation error. However, from qualitatively looking at the eye crop pictures, it appears that only P1 looked at the bottom of the screen while P2 didn't seem to pay attention to the dot. Further, P1's eyes appeared partially closed as they looked down at the bottom of the screen during task performance. In contrast, the eye crop example in Supplementary Fig. 1c is for another random participant who was looking at the bottom portion of the screen. In this case, the eyes were clearly visible and the error was much lower (0.4cm). Overall, when Ps looked down, most of their eye crops resembled Supplementary Fig. 1a, resulting in higher errors.

Downward gaze is a difficult problem, both for our eye tracker and for Tobii glasses (see Supplementary Fig. 2a). One potential workaround may be to use the phone in landscape mode, which minimizes downward gaze. To test this, we collected additional data from 12 participants. As shown in Supplementary Fig. 2b, our model achieves similar accuracy on the landscape mode as for portrait (0.55 ± 0.08cm). The landscape mode may be better suited especially for studies related to images and videos (where landscape is the common mode for viewing). We plan to investigate this further in future work.

While the paper focused on Pixel 2 XL phones, we found that personalization helps across devices. As seen in Supplementary Fig. 3a, personalization led to low errors of 1cm across different iPhones (iPhone5, 5c, 5s, iPhone 6, 6s, 6Plus) from the GazeCapture dataset[1]. Since our study on Pixel 2 XL phones focused on the frontal headpose and near distance, we restricted the iPhones dataset to similar settings to enable a clean comparison. In particular, we limited to those Ps whose tilt / pan / roll was within ±10 degrees, and whose face covered at least 50% of the camera frame. This resulted in at least 5-9 users per device. Due to the higher diversity in headpose and distance in this dataset, we found lower accuracy numbers for iPhones compared to ours (mean ± s.e.m. error of 1.12 ± 0.07cm for iPhones vs. 0.46 ± 0.03cm for Pixel 2 XL).

We further analyzed the effect of headpose and distance on accuracy for the GazeCapture dataset (combining all devices). As seen in Supplementary Fig. 2c-f, the best performance was achieved for near frontal headpose and shorter distance to the phone (where the eye region appeared bigger), and accuracy decayed with increasing pan/tilt/roll, or as participants moved further away from the phone. As mentioned earlier, the accuracy on GazeCapture dataset (Supplementary Fig. 2c-f) is slightly worse than the ∼0.5cm in our study (Fig. 1a-b), since the former consisted of diverse headpose/distances (e.g., zero roll angle still includes diverse pan/tilt/distances) while our study focused on the near frontal headpose with shorter viewing distance.

Comparison with Specialized mobile eye trackers: Supplementary Figure 4a shows the error distribution across 13 Ps in study 1 for the fixed device

stand setting. As mentioned in the paper, there was no significant difference in error at the population level. 6 / 13 Ps showed significantly lower error on Tobii than ours, while 1 / 13 Ps showed the inverse and rest did show any significant difference. Note that the above excludes 4 Ps that were removed due to >1cm error on Tobii (see Supplementary Fig. 4b).

Supplementary Figure 4c shows the error distribution across 13 Ps in study 1 for the handheld setting. 5 / 13 Ps showed significantly lower error on Tobii than ours, while 3 / 13 Ps showed the inverse and rest did show any significant difference. Note that the above excludes 7 Ps that were removed due to >1cm error on Tobii (see Supplementary Fig. 4d).

Head mounted eye trackers like Tobii glasses estimate gaze in the world centered coordinates, which poses additional challenges for quantitative analysis of gaze on screen. Mapping gaze from the world to the phone screen coordinates is time-consuming – the in-built auto-mapping step is challenging and error prone, and often needs manual intervention. In comparison, since our method estimates gaze directly on the phone screen coordinates, it eliminates the mapping step, reduces end-to-end error, and enables easier quantitative analysis of gaze on screen.

Although the exact reason for high errors on Tobii for some participants (after auto-mapping) is unknown, there are a few factors that could cause the auto-mapping algorithm to fail. The first set of reasons is participant related. Some participants had hooded eyes. Other may have looked under the Tobii glasses frame, that led to partially occluded pupil. Certain facial features may have resulted in difficult calibration, that led to unstable sampling. The second set of reasons are stimulus related. While we used QR code like fiducial markers on the corners of the screen to aid in robust recognition, perhaps a richer stimulus with complex images may have enabled automapping to work better. The target may have been close to the edge of the screen. Environmental lighting may have been too intense or the phone screen may have been too bright. The third set of reasons is task related. In the hand-held setting, user's hand may have covered a portion of stimulus patterns on screen. Phone screen may have been held at an angle relative to Tobii's scene camera.

Oculomotor tasks: Study 2 consisted of 6 blocks of 3-5 minutes each, separated by 1 minute of rest. Each block consisted of sub-blocks that could consist of prosaccade, smooth pursuit or visual search tasks in randomized order. Details below.

Supplementary Figure 5 is similar to Fig 3c-e and shows the smooth pursuit box task, and related results. Ps performed the box tracking task well with a low mean error of 0.40cm ([25th, 95th] percentiles were [0.32, 0.56]cm respectively).

Visual search: Two types of visual search tasks were used in our study – one for color intensity and the other for orientation. For the former, participants were presented with a set of green circles on an otherwise blank screen (colored in black). One of the circles (the target) was displayed in the same color, but with a different intensity than the rest. For the orientation task, a set of vertical bars

was shown, with the target appearing in a different orientation from the rest. In both cases, participants were instructed to find the odd item in the display and tap on its screen location as soon as they found it. Items were displayed at an eccentricity of 576 pixels (6.21° viewing angle). Additional random jitter of pixels drawn from a normal distribution (with zero mean, σ=30px) was added to each item's location.

The visual search tasks for color intensity contrast consisted of two sub-blocks of ten trials each. Across trials, target intensity was randomized across four values (from the smallest (1) to the largest (4) color intensity contrast). Similar setup was used for visual search tasks for orientation contrast. In each sub-block, we fixed the distractor orientation at 90° and varied target orientation contrast (difference in orientation between target and distractor) randomly between 5, 10, 15, or 20° across trials.

To test the effects of set size, we focused on orientation contrast. For each target orientation contrast (7, 15, 75°), we varied the set size between 5, 10 and 15 items in the display. Ps performed two sub-blocks, with twenty trials per sub-block. Orientation contrast and set size were varied randomly between trials.

For each visual search task, we computed the number of fixations and the total fixation duration taken to find the target in the display. Results are reported in Fig 4 in the paper.

Reading comprehension: Sample passages and the questions can be found along with the source data files for this manuscript.

Each trial consisted of 3 time segments: 1) initial passage reading, 2) reading and answering the first question; 3) reading and answering the second question. #2 and #3 may involve scrolling back and forth between reading the passage and question. The order of the factual question was randomized across the 10 tasks, such that an equal number of tasks had factual as the first question vs. second. Analysis of factual tasks was restricted to the "factual" time segment – the time from when the factual question was first seen, to when it was answered (#2 or #3 depending on whether the factual task appeared first or second). As seen in Fig 6 in the paper, we found that as task difficulty increased, the number of fixations on the passage taken to find the answer increased and the %total fixation duration on the relevant portion of the passage decreased.

Note that for Fig 6, since the relevant and irrelevant portions of the passage varied in length, we normalized the total fixation duration on the relevant portion by its height (similarly for the irrelevant portion). This enabled a clean comparison of total fixation duration per pixel between relevant and irrelevant portions in the passage.

We tested whether the first time segment of initial passage reading added any value to detecting task difficulty. However, we found that including the time for initial passage reading in addition to the "factual" time segment diluted the results. As seen in Supplementary Fig. 6, none of the metrics (time on answer, number of fixations, %total fixation duration on relevant portion of the passage

normalized by its height) showed any significant variation with task difficulty. We found that %total fixation duration per pixel was higher for the relevant portion than irrelevant when Ps answered correctly (54.64 ± 1.27% vs. 45.36 ± 1.27%, $t(136) = 3.66$, $p = 3x10^{-4}$), though the effect was diluted compared to Fig 6a (where Ps spent 62.29 ± 3.63% on the relevant portion).

In Fig 9, we considered %incorrect as the measure of task difficulty. One shortcoming of this measure is that it does not take the time to answer into account, e.g., a difficult question may be answered correctly if Ps spend enough time reading the passage. Hence, we considered an additional measure of difficulty computed as the ratio of time to answer the question over the %correct. This measure of difficulty is high when Ps take a long time to answer and have low accuracy. Similar to Fig 9e-f, as seen in Supplementary Fig. 7, we found that the number of fixations on the passage increased significantly with this second measure of difficulty. The %total fixation duration on the relevant portion decreased significantly with difficulty, indicating that Ps spent more time looking at the irrelevant sections on difficult tasks.

Natural images: We analyzed the mobile and desktop gaze heatmaps to determine what fraction of images showed significant differences (i.e., low pixel- or object-level correlation). The median pixel-level correlation was high (0.75), with 25th and 75th percentiles at [0.69, 0.80]). Even the least similar images (bottom 5th percentile) had a reasonable correlation of 0.58. Similarly, the median object-level correlation was high (0.95), with 25th and 75th percentiles at [0.89, 0.98]), and even the least similar images (bottom 5th percentile) had 0.65 correlation. This shows that even the least similar mobile and desktop gaze heatmaps were not very different from one another.

Manual analysis of the least similar images (corresponding to the 5th percentile) showed that most differences are due to two reasons, explained below. First, as shown in the first four rows of Supplementary Fig. 8, small objects like fine text, cards and badges tend to draw attention on the large desktop display (third column), however, they appear tiny on the mobile device, hence don't attract much attention on the phone (middle column). Instead larger objects like faces appear more salient on the phone.

For example, in Supplementary Fig. 8-row 1, the gaze heatmap on the phone is focused on the face, while the one on desktop is focused on the fine text on the cover of the book. Similarly in Supplementary Fig. 8-row 2, the mobile heatmap is mostly focused on the face and around bigger objects like the monitors. In contrast, the gaze heatmap on the desktop shows attention hotspots on smaller objects like the fine text on the badge, on the TV screen (on top of the image), and near the bottom-right of the image (which says "Kennedy Space Center"). In Supplementary Fig. 8-row 3, the mobile heatmap is mainly focused on the faces of the players, while the one on desktop is focused on smaller objects like the cards in the hands of the players, the white brand name on the black shirt and the text to the bottom left of the image. Finally, in Supplementary Fig. 8d, the mobile heatmap is focused on the face of the bird, the big glass and around

the center of the laptop monitor; while the one on desktop is focused on smaller objects like the brown keychain on the table, the red box at the bird's feet and the text on the top-right of the laptop (white text, "Touch screen A/V controls", against a black background).

The second type of difference is when the image consists of several small objects (Supplementary Fig. 8, bottom 3 rows). While each of the small objects becomes a separate attention cluster on the desktop gaze heatmap, we find a combined large attention cluster near the centroid of these objects on the mobile heatmap. This is because given the small size of the phone screen, fixating near the centroid of these objects already enables the participants to view the nearby objects in the periphery without requiring multiple fixations on each. In contrast, the large viewing angle on desktop calls for multiple fixations to see the different objects.

Supplementary Figure 9 shows the mobile and desktop gaze heatmaps for a larger blur width corresponding to 1° viewing angle on mobile (67px). The mobile and desktop heatmaps were found to be qualitatively similar, covering similar objects in the image. Even for the least similar heatmaps, we found that similar objects were fixated across both mobile and desktop, though the relative densities were shifted. From a quick glance at the least similar heatmaps, it appears that fixations on small mobile screens was biased towards faces (see first and third rows in Supplementary Fig. 9 for Least Similar) and large text (see fourth row). Quantitatively, as seen in Table 1, we found that both pixel and object level correlations were high for mobile and desktop fixation heatmaps, with Pearson's correlation of >0.8 (higher than baselines such as shuffled desktop or center bias).

Overview of the personalized gaze estimation system: As discussed in the Methods section of the manuscript, the personalized gaze estimation model presented here (see Supplementary Fig. 10) consists of a multilayer feed-forward convolutional neural network (CNN) model[1,2] (hereafter, referred to as the base model) followed by a shallow Support Vector Regression (SVR) model (hereafter, referred to as the personalized model) that is fitted at a user-level to build a high-accuracy personalized model. All models were implemented using the Tensorflow and scikit-learn open-source libraries. Model training took place on dedicated machine learning hardware: Google Tensor Processing Units.

Base model architecture: The inputs to the base model are the left/right eye images, and the eye corner landmark features. The inputs are processed by the CNN and the fully connected (FC) layers and a regression head outputs two numbers for the x- and y-location of gaze on the phone screen. Schematics of the base model architecture is shown in Supplementary Fig.11 along with the details of the network architecture.

Data generation, augmentation & training: The first stage in the gaze model prepares the dataset and trains the base model described previously using a

subset of the publicly available MIT GazeCapture dataset[1]. Specifically, only images in the dataset that came from phones in the portrait screen orientation were used for pre-training as our user studies had a similar device and screen orientation.

The dataset for base model training is prepared as a tf.Example protocol buffer. First, the full frame image is passed through a face detector built on MobileNets with the SSD detector[3] which provides the face bounding box and the eye center landmarks, that are used to generate the eye crops. The eye crop inputs are 128 x 128 x 3 pixels and are standardized by subtracting the mean and dividing by the standard deviation of the pixel intensities in a channel-wise manner. Additionally, the left eye image is flipped left-to-right so that the weights of both eye streams can be shared more easily. The landmark features are computed by concatenation of two dimensional eye corner landmarks (x, y) that amounts to a $\mathbb{R}^8$ vector (4 x 2 floating point numbers) for the two eyes. During training, the eye bounding box and eye corner landmark locations are randomly jittered. This jittering of eye regions makes the model more robust to small changes in lighting, camera movement, noise, or face/eye landmark detection errors. The data augmentation, training and model hyperparameters for the base model are listed below.

- Neural network weight-related params:
  - weights of the network are initialized at random using the tf.keras default weight initializers
  - weight regularization: Disabled
  - conv dropout prob: 0.02
  - fully-connected dropout prob: 0.0 (except FC4 where dropout = 0.12)
- Batch normalization params (see tf.keras.layers.BatchNormalization):
  - batch normalization: Enabled
  - moving average momentum = 0.9
- Learning rate schedule params (see tf.keras.optimizers.schedules.ExponentialDecay):
  - initial learning rate: 0.016
  - decay steps: 8000
  - decay rate: 0.64
  - decay type: 'staircase'
- Optimizer (see tf.keras.optimizers.Adam):
  - type: Adam with $\beta_1 = 0.9$, $\beta_2 = 0.999$, and $\epsilon = 1\text{e-}7$
- Model training params:

  - batch size: 256
  - train steps: 30000

- Loss function:
  - mean euclidean distance error defined

- Data augmentation:
  - random eye cropping: Enabled (see tf.image.random_crop)

Base model finetuning: The next stage of the gaze estimation pipeline finetunes the pre-trained base model using the user study calibration data. Specifically, every study has two types of calibration data: dot calibration for eval and smooth pursuit calibration for training. For this stage, only the smooth pursuit calibration frames belonging to all participants in the study is provided as the training dataset. During fine-tuning, all the layer weights of the pre-trained base model are allowed to be updated until the model converges. The data is pre-processed and the model is re-trained (with the weights of the network are initialized from the pre-trained model) using the same model training parameters as described previously for the base model.

User personalization: After the base model is trained and fine-tuned on the user study calibration data, the next stage extracts a high-level representation from the fine-tuned base model. To this end, we use the fine-tuned base model's penultimate layer output as the feature representation.

The final stage of the gaze estimation pipeline enables a per-participant personalized gaze estimation. In this stage, we first extract the feature embedding of the fine-tuned base model using the calibration frames belonging to a particular participant. We then use the extracted features (from the smooth pursuit calibration task) as the input and the ground truth gaze target as the output to fit a SVR model for that participant and time block. The corresponding dot calibration data in the same time block for the participant is used as the test set for model evaluation. We use the scikit-learn SVR library for fitting this lightweight regression model. The data augmentation, training and model hyperparameters for the personalized model are listed below.

- Data pre-processing params: Filter out calibration task frames where the eye may be in a saccade
  - Dot calibration: Keep frames with time since calibration dot onset in [800, 1500] ms
  - Pursuit calibration: Keep frames with time since calibration dot onset >1300 ms (for ∼30 sec)

- SVR params (see sklearn.svm.SVR):
  - kernel: 'rbf'

– C: 20.0

– gamma: 0.06

Model fitting method:

– Per participant per task block SVR model

– Training is based on a leave-one-task-out setup where pursuit calibration data is used for model training and dot calibration data is used for model evaluation

Gaze inference: During inference, the pre-trained base model and the regression model are applied in sequence to an image to generate the final, personalized gaze estimate.

Supplementary References

[1] Kyle Krafka, Aditya Khosla, Petr Kellnhofer, Harini Kannan, Suchendra Bhandarkar, Wojciech Matusik, and Antonio Torralba. Eye tracking for everyone. *2016 IEEE Conference on Computer Vision and Pattern Recognition*, 2016.

[2] Junfeng He, Khoi Pham, Nachiappan Valliappan, Pingmei Xu, Chase Roberts, Vidhya Navalpakkam, and Dmitry Lagun. On-device few-shot personalization for real-time gaze estimation. In *2019 International Conference on Computer Vision: Workshop on Gaze Estimation and Prediction in the Wild*, 2019.

[3] Wei Liu, Dragomir Anguelov, Dumitru Erhan, Christian Szegedy, Scott Reed, Cheng-Yang Fu, and Alexander C Berg. Ssd: Single shot multibox detector. In *European Conference on Computer Vision*, pages 21–37. Springer, 2016.
